# Comorbidity of attention deficit hyperactivity disorder with personality disorders in homeless people

**DOI:** 10.1186/1756-0500-7-916

**Published:** 2014-12-16

**Authors:** Carlos Salavera, José Luis Antoñanzas, Juan Carlos Bustamante, José Carrón, Pablo Usán, Pilar Teruel, Carmen Bericat, Lucía Monteagudo, Soledad Larrosa, José M Tricás, Orosia Lucha, Raquel Noé, Laurane Jarie, Raquel Cerra

**Affiliations:** OPIICS Research Group, University of Zaragoza, C/Domingo Miral s/n, 50009 Zaragoza, Spain; Faculty of Health Sciences, University of Zaragoza, C/Domingo Miral s/n, 50009 Zaragoza, Spain; Faculty of Education, University of Zaragoza, C/Pedro Cerbuna, 12, 50009 Zaragoza, Spain; Physiotherapy Research Unit, Faculty of Health Sciences, University of Zaragoza, C/Domingo Miral s/n, 50009 Zaragoza, Spain; Faculty of Social Sciences and Work, University of Zaragoza, C/Violante de Hungría s/n, 50009 Zaragoza, Spain

**Keywords:** Hyperactivity, Personality disorders, Comorbidity

## Abstract

**Background:**

Attention deficit hyperactivity disorder (ADHD) is a condition that begins in childhood but can continue into adulthood, and may be the cause of many disadaptive behaviors, as in the case of homeless people, who often display a high incidence of personality disorders. The goal of this study is to analyze the comorbidity of ADHD with axis II disorders in a Spanish homeless population.

**Results:**

The outcomes show high comorbidity between these two kinds of disorders, and that the prevalence of axis II disorders is higher among people with ADHD than among the general population.

**Conclusions:**

From these results we can draw the conclusion that in homeless people ADHD in childhood continues into adulthood, when it is very often observed together with personality disorders. Finally, the implications of this study both for clinical practice and for future lines of research are discussed.

## Background

In recent years (2005–2012) the homeless population has increased 132% in Spain and died in our country a homeless person every five days [1]. Last data reported that more than 22.000 people in homelessness situation were treated in health centers [1]. Currently homelessness is on the increase due to high levels of unemployment and lack of job opportunities for young people. This situation gives rise to extremely high rates of unemployment amongst people aged between 25 and 35, the percentage of unemployment in this segment of the population being significantly higher than that of the general population. The homeless are one of the most vulnerable and disadvantaged groups in society, and in Western Europe and North America alone it is estimated that more than one million people are without homes. These people may take refuge temporarily in shelters, although the economic crisis has led to a higher number living in the city streets themselves. They are associated with traumatic ruptures and economic, family and social problems, as well as a low quality of life and high rates of physical and psychic diseases. They suffer obvious mental deterioration as a result of living in the streets, and this is considered the highest level of social exclusion in a modern society [2]. A number of studies have found higher rates of serious mental disorders in homeless people [3]. The most common mental disorders are psychosis, drug dependence, depression and personality disorders.

Attention deficit hyperactivity disorder (ADHD) is a development disorder with its origin in childhood but which can persist in adulthood [4, 5]. The functional anomalies associated with ADHD include poor work and school performance [6].

It is estimated that about 3-5% of children and nearly 4% of adults present ADHD [7, 8]. A meta-analysis of follow-up studies concluded that in up to 65% of children with ADHD, symptoms and anomalies can persist into adulthood [9]. To diagnose ADHD in adults we need to determine that the adult suffered ADHD in childhood and meets the current criteria for adult ADHD. This clinical profile might include inattentive symptoms, hyperactive-impulsive symptoms, altered executive functioning, and emotional and behavioral dysregulation [10, 11]. Low frustration tolerance, emotional lability and difficulty managing strong emotions are presented in this population [12]. Adults with ADHD show a high rate of psychiatric comorbidity with mood and anxiety disorders, substance abuse, and personality disorders [13–17].

Symptoms of ADHD in childhood are consistent with those showed by homeless people, who display social, emotional and personal instabilities which influence both their diagnosis and prognosis. Moreover, the prevalence of organic and mental disorders is much higher in this population than in the population at large [18, 19]. In spite of this, the presentation of ADHD in childhood and adulthood in the homeless population has not been extensively studied, and this may affect its prognosis. The evaluation of its comorbidity is very important in this case, due to the high rates of personality disorders present in this particular population [20].

The goal of this study is to analyze the comorbidity of ADHD and personality disorders in homeless people.

## Methods

### Subjects

Most homeless people in Spain receive assistance from various public-funded municipal bodies, and it is very difficult to determine the percentage of this population relative to the general population. Our study was carried out in a city of 700.000 inhabitants. The sample consisted of homeless people (N = 196) taking part in a process of psychosocial insertion at a special centre of Zaragoza (Spain), where they received psychological and social support and occupational training. The participants in the study were selected from among 203 people involved in the insertion process and were chosen according to the following criteria: a) condition of homelessness; b) having stayed for more than two months in the centre; c) voluntary participation in the study; and d) a long enough stay for the completion of the study. All the participants provided written informed consent prior to participating in the study. This study was approved by the ethics committee of Department of Psychology and Sociology at the University of Zaragoza, which complied with the ethical requirements of the Declaration of Helsinki.

Given the aim of our study a clinical psychologist carried out the assessment process to evaluate the ADHD-related clinical profile of each participant, where they self-reported the existence of ADHD in childhood or a current profile of ADHD in adulthood [10].

### Assessment measurements

- Initial assessment interview. To begin with, a structured clinical individual interview was conducted in order to establish a diagnosis. At this interview the salient data were collected: age, marital status, educational level, age at which the subject began a transient life, the causes that led her/him to this kind of life, incidence of alcohol or drug abuse.
- MCMI II. Millon Multiaxial Clinical Inventory II [21]. This is composed of 175 true/false statements that reportedly take 25–30 minutes to answer. It provides 10 basic personality scales, three pathological personality scales, six moderately severe clinical syndrome scales and three severe clinical syndrome scales (Cronbach’s alpha = 0.86).
- ASRS. Adult Self-Report Scale Version 1.1 (ASRS v.1.1) [22]. This is an 18-item questionnaire for measuring current ADHD symptoms in adults. Each item is a question about symptoms presentation in the 6 months prior to the assessment, answered using a 5-option Likert scale (Cronbach’s alpha = 0.88).
- WURS. Wender-Utah Rating Scale [23]. For assessment of ADHD in childhood; a 25-item self-applied questionnaire. The subject answers questions about her/ his behaviour in childhood using a 4-option Likert scale (Cronbach’s alpha = 0.90).

### Procedure

The participants’ profile up to the present was analyzed using an initial structured interview, reflecting mainly sociodemographic data and personal history as a homeless person. The statistical analysis of the data was carried out using the statistical program SPSS version 21.0. A descriptive analysis was performed (maxima, minima, averages and standard deviation) for each of the variables. In all cases the significance level used was 5%. Crossed variables and bilateral correlation analyses were also carried out. All the tests were applied and corrected by the clinical psychologist in charge of the research.

## Results

### Sociodemographic variables

This section evidences the high proportion of young homeless people included in the integration process (Table 1). 64.79% of the subjects in the process were younger than forty. The most frequent marital status of the subjects was single, at 57.14% (n = 112), while 39.78% (n = 78) were separated or divorced. In schooling, high early school dropout rates were detected. 37.34% (n = 73) of the sample obtained only the high school certificate (up to age 14), and only 18.36% (n = 36) continued studying after completing compulsory education. The commonly early beginning of the transient life was also observed, 42.34% of the subjects having embarked on this kind of life even before the age of 20. The reasons that led these people to their life in the streets were mainly problems with their family of origin (28.57%), addictions (32.14%) and divorce (15.81%).

**Table 1:** **Sociodemographic features of the sample (N = 196)**

		Frequency	Percentage
Age	<30	59	30.10%
	30-39	68	34.69%
	40-49	57	29.08%
	>50	12	6.12%
Marital status	Single	112	57.14%
	Married	3	1.53%
	Divorced	35	17.85%
	Separated	43	21.93%
	De facto union	3	1.53%
Schooling	Without schooling	3	2.04%
	High School Certificate	73	37.24%
	Primary School	84	42.85%
	Professional School	23	11.73%
	Pre-University Studies	13	6.63%
Age when started living in the street	<20	83	42.34%
	20 a 29	69	35.20%
	30 a 39	40	20.40%
	40 a 49	4	2.04%
Reasons for living in the street	Divorce	31	15.81%
	Problems with family of origin	56	28.57%
	Work problems	21	10.71%
	Addictions	63	32.14%
	Psychological problems	23	11.73%
	Other	2	1.02%

### Personality variables

According to the results obtained from the Millon Multiaxial Clinical Inventory (MCMI-II), 154 of the subjects (78.57% of the sample) showed one or more personality disorders (Table 2). Antisocial (26.4%; n = 37), dependency (22.1%; n = 31), compulsive (26.4%; n = 37) and schizoid (21.4%; n = 30) disorders obtained the highest scores, using a Base Rate (BR) > 84 following more conservative criteria [24]. We should highlight that some subjects’ test results showed that they may have one or more high-scoring subscale.

**Table 2:** **MCMI II scores (N = 196)**

	Minimum	Maximum	Average	S.D.
Schizoid	0	116	58.67	28.23
Phobic	1	103	50.37	29.69
Dependent	0	108	55.29	30.6
Histrionic	5	103	52.50	25.62
Narcissistic	0	106	56.59	25.67
Antisocial	0	124	66.71	28.93
Aggressive	2	121	55.63	28.86
Compulsive	5	118	68.06	27.49
Passive	0	103	42.34	26.3
Self-destructive	2	109	50.91	25.92
Schizotypal	3	117	56.86	27.16
Limit	0	116	48.59	27.26
Paranoid	4	118	61.31	25.59

The number of personality disorders present in each subject was analyzed. Some subjects receiving the homeless insertion treatment showed no personality disorders (16.42% of cases; n = 23), while others in the sample displayed one (22.14%; n = 31), two (12.14%; n = 17) or even three or more (49.28%; n = 69).

### ADHD variables

Results showed that 14.28% of the sample (n = 28) could be diagnosed as suffering from ADHD in childhood, whereas 8.16% of the participants (n = 16) would have a current diagnosis of ADHD. This would imply that 57.14% of the subjects would maintain this diagnosis throughout their lives.

Furthermore, comorbidity of personality disorders and ADHD was studied (Table 3), taking into account both past (WURS) and current (ASRS) diagnoses of ADHD. Whether a diagnosis of ADHD in childhood could determine in any way a personality disorder in adulthood was also analysed. In this sense, we found an association between personality disorders like aggressive, compulsive, self-defeating, borderline and paranoid disorders and the past ADHD diagnoses in childhood. Likewise, we found a significant effect in the association between those personality disorders and current ADHD in adulthood.

**Table 3:** **Comorbidity of ADHD and personality disorders**

		WURS			ASRS		
		Absence	Presence	***x*** ^***2***^	Absence	Presence	***x*** ^***2***^
**Schizoid**	Absence	131	23	68.67	138	16	172.02***
	Presence	33	9		36	6	
**Phobic**	Absence	141	23	60.27	155	9	133.63***
	Presence	23	9		19	13	
**Dependent**	Absence	124	28	69,59	133	19	153.34***
	Presence	40	4		41	3	
**Histrionic**	Absence	149	26	69.39	156	19	183.45***
	Presence	15	6		18	3	
**Narcissistic**	Absence	147	29	53.67	160	16	98.0***
	Presence	17	3		14	6	
**Antisocial**	Absence	124	17	60.49	141	0	168.40***
	Presence	40	15		33	22	
**Aggressive**	Absence	143	22	78.25**	156	9	188.47***
	Presence	21	10		18	13	
**Compulsive**	Absence	126	16	75.91*	126	16	154.71***
	Presence	48	6		48	6	
**Passive**	Absence	157	25	69.13	170	12	173.42***
	Presence	7	7		4	10	
**Self-destructive**	Absence	151	25	66.65*	161	15	169.08***
	Presence	13	7		13	7	
**Schizotypal**	Absence	135	23	64.29	149	9	145.07***
	Presence	29	9		25	13	
**Limit**	Absence	154	20	73.26*	174	0	196.00***
	Presence	10	12		0	22	
**Paranoid**	Absence	139	25	69.02*	151	13	158.72***
	Presence	25	7		23	9	

*p < .05; **p < .01; ***p < 0.01.

Finally, we sought to determine whether suffering from ADHD could lead to the presentation of a personality disorder in adulthood (Table 4). The outcomes in this section indicated a higher correlation between ADHD and personality disorders as the age of the subjects increased.

**Table 4:** **Correlation between ADHD and personality disorders**

	WURS	ASRS
Schizoid	.02	-.01
Phobic	.20^**^	.39^**^
Dependent	-.15^*^	-.22^**^
Histrionic	.06	.05
Narcissistic	.08	.26^**^
Antisocial	.21^**^	.46^**^
Aggressive	.15^*^	.47^**^
Compulsive	-.17^*^	-.10
Passive	.18^*^	.43^**^
Self-destructive	.1	.41^**^
Schizotypal	.19^**^	.38^**^
Limit	.29^**^	.68^**^
Paranoid	.04	.34^**^

**The correlation is significant at a level of 0.01 (bilateral).

*The correlation is significant at a level of 0.05 (bilateral).

## Discussion

Previous studies show a 4% occurrence of ADHD in adults, although some authors consider that this disorder is underdiagnosed and consequently wrongly treated [25]. In common with existing studies [9, 13, 26], in the present study it was found that nearly two out of every three subjects diagnosed in childhood were still diagnose d with this disorder on reaching adulthood. It could be affirmed that the comorbidity of ADHD in adults is more the norm than the exception [27], and that this factor may lead to diagnostic and therapeutic failure.

In our research strong correlations between the outcomes obtained both with ASRS and MCMI-II were observed, although the first measures axis I disorders such as inattention, hyperactivity and impulsivity [28], and the second axis II disorders such as complex behaviour patterns [29]. This would mean that the ASRS measures symptoms common to personality disorders, especially to the disruptive personality. In the case of the results obtained from the WURS questionnaire, their correlations with the MCMI-II were lower than in the case of MCMI-II with ASRS. This would mean that as age increases, even when the incidence of personality disorders decreases, the comorbidity of ADHD and personality disorders increases [30]. It remains to clarify whether ADHD acts as a moderator or mediator in the subsequent presentation of personality disorders, along the lines suggested [31]; however both disorders fit into a pattern of comorbidity [20].

Not every personality disorder showed a positive correlation with ADHD. The correlations observed with both the dependent and the compulsive disorders were negative. The correlation was stronger with the more severe disorders, both impulsive spectrum disorders with "fight response" (antisocial, negativistic and limit) and schizophreniform spectrum disorders with "flight response” (schizotypal, self-destructive and avoidant). These results coincide with those obtained in previous studies [32].

## Conclusions

According to the results of this study where the study population demonstrate an early beginning of transient living, the homeless people examined displayed more significant psychopathological symptoms, both in ADHD and the personality disorders, than the general population, in addition to the correlation between psychological and personality disorders [33, 34]. Homeless people show difficulties in achieving goals and maintaining an adequate level of performance at work, as well as in developing satisfactory interpersonal relationship [35]. Our results showed how difficult is for this population to establish and maintain a steady relationship. These difficulties are probably connected with the academic and labour problems displayed by people with ADHD, and may demonstrate the connection between both pathologies.

These findings have useful clinical implications due to determine a better comprehension of specific clinical profile in homeless people for setting adjusted intervention programs. However, the sample size was relatively small from a statistical perspective. Moreover, other limitation could be related with the fact that we did not include a control group in the sampling process. This methodological approach could give more consistency to the empirical evidence showed in our study. Future studies will overcome these limitations, and are required in order to identify the specific profiles and evolution of attention deficit hyperactivity and personality disorders in homeless population.

In future lines of investigation, the presence of other clinical manifestations in conjunction with ADHD should be studied, since this causes confusion in the therapeutic approach and makes treatment of these patients more difficult. Furthermore, it is possible that the comorbidity of ADHD with other disorders has different clinical subtypes, phenotypes or other phenotype expressions that should be studied to achieve a better understanding of this pathology.

## Abbreviations

ADHD: Attention deficit hiperactivity disorder
ASRS: Adult self-report scale
INE: Institute national of stadistical
MCMI II: Millon multiaxial clinical inventory II
OPIICS: Observatory for investigation and innovation social sciences
WURS: Wender-Utah rating scale.
